# Improving prediction of 12-months suicidal attempts in bipolar disorder: a machine learning study

**DOI:** 10.1192/j.eurpsy.2024.719

**Published:** 2024-08-27

**Authors:** A. Pigoni, G. Delvecchio, C. Pini, L. Cirella, C. Prunas, N. Turtulici, L. Squarcina, P. Brambilla

**Affiliations:** ^1^Department of Neurosciences and Mental Health, Fondazione IRCCS Ca’ Granda Ospedale Maggiore Policlinico; ^2^Department of Pathophysiology and Transplantation, University of Milan, Milan, Italy

## Abstract

**Introduction:**

Bipolar disorder (BD) is a recurrent disorder, causing functional impairment and **raised mortality, particularly due to suicide**. However, the difficulty in predicting suicidal behaviors relies in the **lack of clear biomarkers**.

Machine learning (ML) has emerged as a promising tool to enhance suicidal prediction. However, most **ML studies focused on lifetime attempts**, without having a predictive time window, and **did not employ time-dependent variables**. Moreover, most studies lie on cross-sectional databases, without including more than one time-point.

**Objectives:**

First, we aimed to predict 12-months **suicide attempts** in a naturalistic sample of BD patients, using clinical and demographic data.

Second, we aimed to improve the prediction by including information from intermediate visits (1, 3, and 6 months), mimicking more closely the clinician’s way of thinking and the multiple observations a patient receives.

**Methods:**

A sample of 163 BD patients (53% females, mean age 44.7, SD 15.3) were recruited.

Based on EHR, **56 clinical and demographic features were extracted**, **including hospitalizations, suicidal behaviors lifetime and in the last 12 months**, along with comorbidity, family history, work, and therapies. **Patients were followed up for 12 months.**

Support Vector Machine (SVM) was used to differentiate subjects who attempted suicide versus those who did not **in a 12-month time window**, within a repeated nested Cross-Validation. The **SVM was optimized weighting the hyperplane** for uneven group sizes.

Then, **we repeated the analysis including information from intermediate visits** (1, 3, 6 months after the first contact). **For each visit, we created a composite score** based on current therapy, new admissions, and ER presentations. To avoid circularity, all the information (ER, admission etc.) related to a suicide attempt were not included.

**Results:**

During the 12-months follow-up, **9.8% of patients attempted suicide**. The results from the 12-months suicide prediction model obtained an Area Under the Curve of 0.71(with a Balanced Accuracy (BAC) of 68%).

**After incorporating the composite scores based on intermediate visits in the model, the prediction raised** to an Area Under the Curve of 0.78 (BAC 73%), suggesting that including intermediate visits is a valid method to improve prediction.

The features that contributed the most to the prediction were **the composite score at 6-month visit, lifetime number of suicide attempts, suicide attempts in the last 12 months, substance of abuse** (other than cannabis), and antipsychotics.

**Conclusions:**

ML proved a good prediction accuracy for suicide in a 12-months time window, and the prediction was improved by including data from intermediate visits. **The model showed the importance of time-dependent features, such as attempts in the last 12 months.** Our analysis might help in identifying early clinical risk factors and underlies **the importance of multiple evaluations in populations at risk**.

**Disclosure of Interest:**

None Declared

